# *In Vitro* Study of Synergic Effect of Cisplatin and Low Molecular Weight Heparin on Oral Squamous Cell Carcinoma

**DOI:** 10.3389/fonc.2020.549412

**Published:** 2020-11-18

**Authors:** Fabio Camacho-Alonso, T. Gómez-Albentosa, R. E. Oñate-Sánchez, M. R. Tudela-Mulero, M. Sánchez-Siles, Francisco J. Gómez-García, Yolanda Guerrero-Sánchez

**Affiliations:** ^1^ Department of Oral Surgery, University of Murcia, Murcia, Spain; ^2^ Private Practitioner, Murcia, Spain; ^3^ Department of Dentistry for Special Patients, University of Murcia, Murcia, Spain; ^4^ Department of Oral Medicine, University of Murcia, Murcia, Spain; ^5^ Department of Human Anaothomy and Psicobiology, University of Murcia, Murcia, Spain

**Keywords:** cisplatin, low molecular weight heparin, oral squamous cell carcinoma, enoxaparin sodium, *in vitro* cell line

## Abstract

**Objectives:**

To evaluate the possible synergic effect of cisplatin and low molecular weight heparin (LMWH) on oral squamous cell carcinoma (OSCC).

**Materials and Methods:**

Cisplatin and enoxaparin sodium, alone or in combination, were administered at doses of 1, 2, 4, 8 and 10 µM and 0.1, 0.5, 1, 5, 10, 50, and 100 µg/ml, respectively, to the H357 human OSCC line. The effects on cell viability and apoptosis were evaluated after 24, 48, and 72 h and on cell migration after 18 and 24 h.

**Results:**

10 µM concentration of cisplatin produced the greatest decrease in cell viability, with significant differences at 24 (*p*=0.009), 48 (*p*=0.001) and 72 h (*p* = 0.003); the 100 µg/ml dose of enoxaparin produced the greatest decrease in cell viability but without significant differences (*p*>0.05). When different concentrations of cisplatin and enoxaparin were combined, it was found that 100 µg/ml enoxaparin sodium produced the greatest synergic effect on cell viability reduction. In analyses of apoptosis and cell migration, it was found that the combination of cisplatin at 8 or 10 μM and 100 μg/ml enoxaparin produced a higher rate of apoptosis at 24, 48, and 72 h and a greater reduction in cell migration at 18 and 24 h.

**Conclusions:**

A combination of cisplatin and enoxaparin sodium shows a synergic effect that reduces cell viability and cell migration capacity and increases the apoptosis of human OSCC cells.

**Clinical relevance:**

Enoxaparin may be beneficial in chemotherapy for patients with OSCC; this finding requires further clinical and laboratory investigation.

## Introduction

Cancer is the main cause of death in both the developed and the developing worlds. It is predicted that numbers of death resulting from cancer will grow as populations and life expectancy increase, especially in the developing world where 82% of the world’s population is located. In the least developed countries, lifestyle habits that constitute risk factors for developing cancer are spreading – smoking, alcohol consumption, a nutritionally poor diet (low consumption of fruit and vegetables), physical inactivity (obesity), and changing reproductive habits (fewer births, later in life) – and the numbers of cases of cancer have increased (1). Squamous cell carcinoma of the head and neck (SCCHN) is the fifth most common form of cancer and the sixth main cause of cancer mortality in the world (2), with approximately 600,000 new cases diagnosed worldwide each year (3). Oral squamous cell carcinoma (OSCC) is the most common SCCHN and represents approximately 3% of new cases of cancer diagnosed (4). Current OSCC treatment includes surgery, radiotherapy and chemotherapy. But long-term survival remains low. In fact, the survival rate of patients with OSCC beyond 5 years is about 50% (5).

Conventional chemotherapy for OSCC is based on cisplatin (cis-diammine-dichloro-platinum), the first of a family of drugs that currently include carboplatin, oxaliplatin, satraplatin, and picoplatin. Among medical cancer treatments, chemotherapy with cisplatin has the greatest impact and its introduction has changed the therapeutic management of a range of tumors over the last 40 years. These include cancers of the bladder, breast, lung, lymphomas, testicles, ovaries (6), as well as SCCHN including OSCC (7). Cisplatin’s mechanism of cytotoxic action on cancer cells is based on inducing apoptosis and cell cycle arrestthrough its interaction with DNA that leads to the formation of cisplatin-DNA adducts, which activate multiple signaling pathways see (8) and (9). In comparison with other types of anticancer cell, cisplatin enters cells relatively slowly. This is regulated by various factors such as sodium and potassium ions, pH regulation, and the action of transporters (10). Before attaching to DNA in cell cytoplasm, cisplatin activates by replacing one of its two chlorine atoms with water molecules. In this way, it covalently binds to DNA, which produces what are known as DNA adducts. The resulting products can cause damage to the DNA of the carcinogenic cells, blocking their division (by blocking cells in the G2 phase of the cell cycle, the mitotic phase) and leading to cell death resulting from apoptosis (11).

In addition to the adverse effects of this drug (nausea, vomiting, dose- and time-dependent toxicities, in particular nephrotoxicity, cardiotoxicity, neurotoxicity and ototoxicity) (12), there are various routes by which cells can develop resistance to the anticarcinogenic action of cisplatin on OSCC. The molecular mechanisms responsible for cell resistance to cisplatin are complex, and may be related to limited cisplatin entrance into cells, intracellular cisplatin deactivation, increased tolerance by the cells, or even increased cisplatin exit to the cell’s exterior (13). As a consequence, the formation of cisplatin-DNA adducts decreases, reducing cytotoxicity, which results in greater resistance (11). Furthermore, according to theories of cancer stem cell behavior (CSC), tumors organize themselves hierarchically in similar ways to healthy tissue, with a sub-population of CSCs that may be resistant to the chemotherapy administered, and that generate differentiated cancer cells (14). This subpopulation of CSCs was first identified in leukemia and later isolated in solid tumors including breast, brain, lung, liver, prostate, colon, and pancreatic cancers (15–19), as well as SCCHN (20–22). The CSCs express high levels of ATB-binding-cassette (ABC), transporter proteins in numerous drugs that are the cause of resistance to treatment by chemotherapy. Some ABC protein families are responsible for the cytoprotective effect of cancer cells against cisplatin (23–25). For this reason, there is a need to develop new anticarcinogenic therapies.

Low molecular weight heparins (LMWHs) were approved by the US Food and Drug Administration (FDA) in 1998 as an anticoagulant treatment and have been administered satisfactorily ever since (26). More recently, several studies have shown that LMWHs reduce death by cancer in patients with deep-vein thrombosis, and different types of cancer (27–29). Although various clinical studies have shown that LMWHs prolong survival and reduce mortality in patients with advanced solid cancer, the exact mechanism whereby LMWHs exercise their anticarcinogenic action has not yet been determined (30–33). Their anticarcinogenic action is probably produced through an antiproliferative action (due to their anti-angiogenic action) (34–36) and antimetastatic action (37–40). Regarding their antiproliferative action, LMWHs have been shown to exert an anti-angiogenic action that regulates tumoral angiogenesis *via* two paths; on the one hand, by impeding thrombin generation, which inhibits the tissue factor pathway through the release of an endothelial tissue factor (TF) pathway inhibitor (TFPI) (41), and on the other, by inhibiting the formation of Xa factor through the attachment of the antithrombin-herapin complex to this factor (42). Its antimetastatic activity would appear to be related to its capacity for attachment to selectins (mainly P- and L-selectin), integrins (mainly VLA-4), cytokines, and enzymes such as heparanases that are able to degrade the extracellular matrix and the components of the basal membrane (38–40).

Enoxaparin sodium is an LMWH obtained by an alkaline depolymerization method; it has an average molecular weight of 4.5 kDa, and its anticarcinogenic activity has been studied in cases of pancreatic adenocarcinoma cells, human breast carcinoma cells, human lung adenocarcinoma epithelial cells, glioma cells, melanoma cells (37, 43–47) and against metastasis from brain and colon cancer (48, 49). But its anticarcinogenic action on OSCC, alone or in combination with cisplatin, is unknown.

The aim of this study was to evaluate the possible synergic effect of cisplatin and enoxaparin sodium on OSCC.

## Materials and Methods

### Cell Line

The study used the H357 human OSCC line (European Collection of Cell Cultures), belonging to stage 1 OSCC (T1N0M0) located at the base of the tongue of a male patient. Cells were cultured in Iscove’s Modified Dulbecco’s Medium (IMDM) supplemented with 10% fetal calf serum (FCS), glutamine (2 mM), 0.5 μg/ml hydrocortisone sodium succinate, 1% penicillin, and 1% streptomycin (full medium) at 37°C, in an atmosphere of 95% oxygen and 5% CO_2_.

The medium (IMDM), 3-(4,5-dimethyl-2-thiazolyl)-2,5-diphenyl-2H-tetrazolium bromide (MTT), dimethylsulfoxide (DMSO), cisplatin, and enoxaparin sodium used in the study were supplied by Sigma-Aldrich^®^ (Sigma-Aldrich Chemistry, S.A., Madrid, Spain).

### Drug Preparation

Cisplatin was dissolved in 0.5% DMSO and enoxaparin sodium in phosphate buffered saline (PBS), with 1 mg/ml of cisplatin or enoxaparin sodium being used as a stock solution. The working solutions were diluted with Iscove’s modified Dulbecco’s medium (IMDM). All manipulations with cisplatin and enoxaparin sodium were performed under subdued lighting. The dose range was 1, 2, 4, 8 and 10 µM of cisplatin and 0.1, 0.5, 1, 5, 10, 50, and 100 µg/ml of enoxaparin sodium.

### Cell Viability Test (MTT)

The technique described by Carmichael et al. (50, 51) was used for cell viability quantiﬁcation, adapted to the study’s culture conditions. The cells were cultured at a density of 3,200 cells per well in 96-microwell plates, after which cisplatin or enoxaparin sodium were added at different concentrations (1, 2, 4, 8, and 10 µM of cisplatin and 0.1, 0.5, 1, 5, 10, 50, and 100 µg/ml of enoxaparin sodium), individually or in combination.

At different time points after the start of treatment (24, 48, and 72 h), the medium was eliminated and the cells were incubated with MTT (Sigma-Aldrich Chemistry, S.A.) (1 mg/ml) for 4 h, after which the non-metabolized MTT was discarded and 100 µl of DMSO were added to each well. Absorbance in each well was measured with an enzyme-linked immunosorbent assay (ELISA), using a Multiskan MCC/340P plate spectrophotometer at a reading wavelength of 570 nm and a reference wavelength of 690 nm. Each test was performed in triplicate.

### Apoptosis (Histone/DNA Fragment ELISA)

The ELISA cell death detection kit was used (following the manufacturer’s instructions) to detect apoptosis in cells treated with cisplatin and enoxaparin sodium. Brieﬂy, cells were seeded in 96-well plates at a density of 3,200 cells per well for 24 h, adding the medium containing the two highest concentrations of cisplatin used in the cell viability test (8 and 10 µM) combined with the highest concentration of enoxaparin sodium used in the cell viability test (100 µg/ml). After 24, 48, or 72 h, the cytoplasm inthe control and treatment groups was transferred to the 96-well plate, peridiumed by streptavidin, and incubated with biotinylated histone antibody and peroxidase-tagged mouse anti-human DNA for 2 h at room temperature. Absorbance at 405 nm was measured with EXL-800 type Enzyme-Linked Immunosorbent apparatus. Each test was performed in triplicate.

### Migration (Scratch Wound Healing)

Scratch wounds were generated in confluent monolayers of cells using a sterile 200 µl pipette tip (52). After washing away suspended cells with phosphate buffer saline (PBS), the culture medium was changed and added at different concentrations: the two highest concentrations of cisplatin used in cell viability testing (8 and 10 µM) combined with the highest concentration of enoxaparin sodium used in cell viability testing (100 µg/ml). Migration into the wound space was photographed using an inverted microscope equipped with a digital camera at the time of the initial wound and at time intervals up to 18 and 24 h after wounding. The relative distances between edges of the injured monolayer were obtained by means of pixel counts at a minimum of 10 sites per wound, using MIP-4^®^ image software (CID, Barcelona, Spain) and applying the formula: migration distance = initial distance of free-of-cells space – distance at 18 or 24 h of free-of-cells space (53). Each test was performed in triplicate.

### Statistical Analysis

Data were analyzed using the SPSS version 20.0 statistical software package (SPSS^®^ Inc., Chicago, IL, USA). A descriptive study was made of each variable. The associations between different quantitative variables were studied using one-way analysis of variance (ANOVA) for more than two samples, verifying in each case whether variances were homogeneous. Statistical significance was accepted for *p ≤* 0.05.

## Results

### Effects of Cisplatin, Enoxaparin Sodium, and the Combination of the Two on H357 Cell Viability

**Figure 1:**
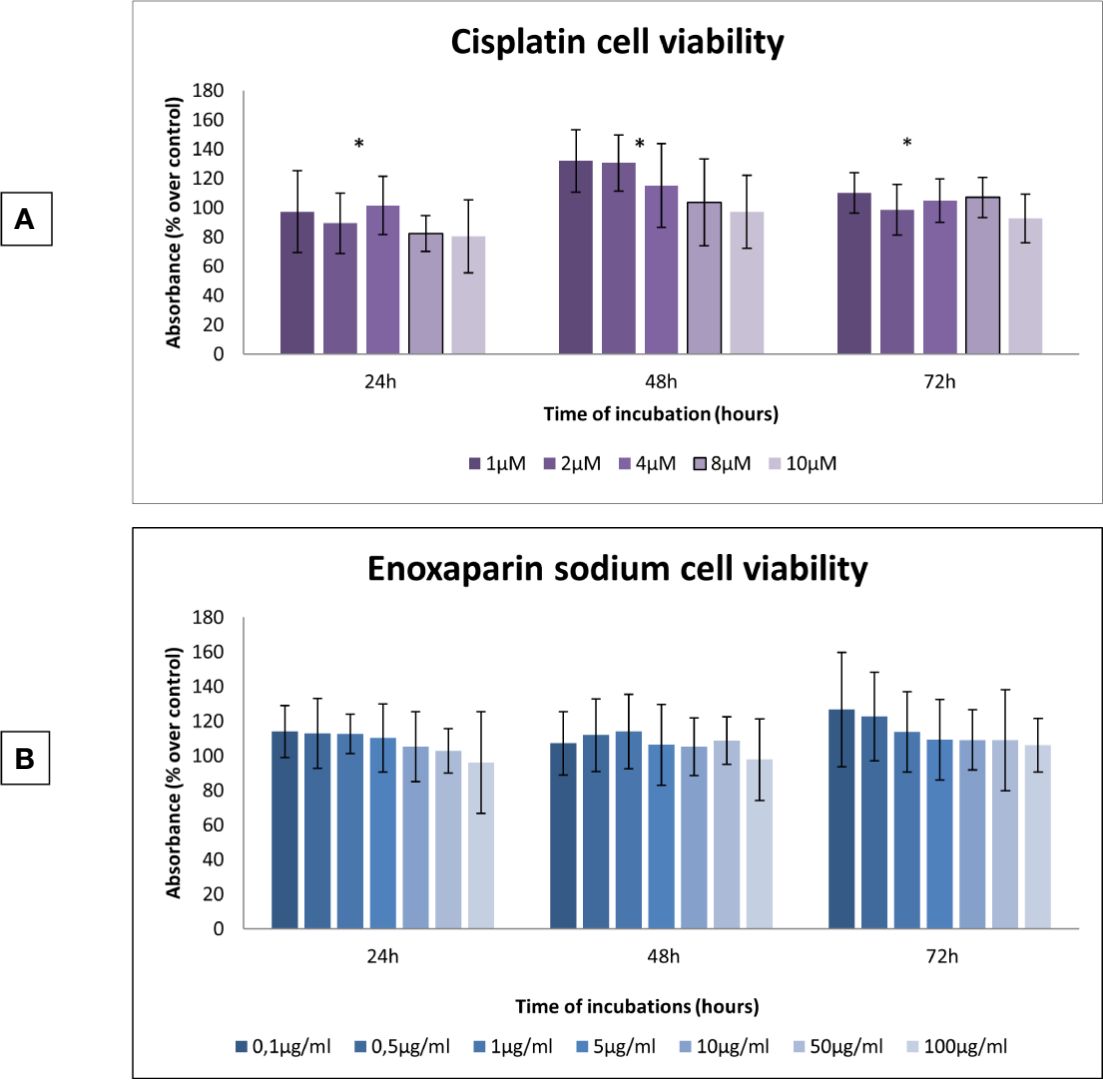
Effects of cisplatin or enoxaparin sodium on H357 cell viability. **(A)** 24 h, *p* = 0.009; 48 h, *p* = 0.001; 72 h, *p* = 0.003. **(B)** 24 h, *p* = 0.215; 48 h, *p* = 0.558; 72 h, *p* = 0.303. * means that there is significative differences at such picture.

At all incubation times (24, 48 and 72 h), it was found that as the dose of cisplatin increased, OSCC cell viability decreased. The 10 μM cisplatin concentration produced the greatest reduction in cell viability, with statistically significant differences at 24 h (*p*=0.009), 48 h (*p*=0.001), and 72 h (*p*=0.003) (**Figure 1A**). When the effect of enoxaparin sodium on cell viability was analyzed at 24, 48, and 72 h incubation, it was found that as the dose of LMWH increased, cell viability decreased, with the greatest reduction seen with the 100 μg/ml dose of enoxaparin sodium, although without statistically significant differences at 24 h (*p*= 0.215), 48 h (*p*=0.558), or 72 h (*p*=0.303) incubation (**Figure 1B**).

**Figure 2:**
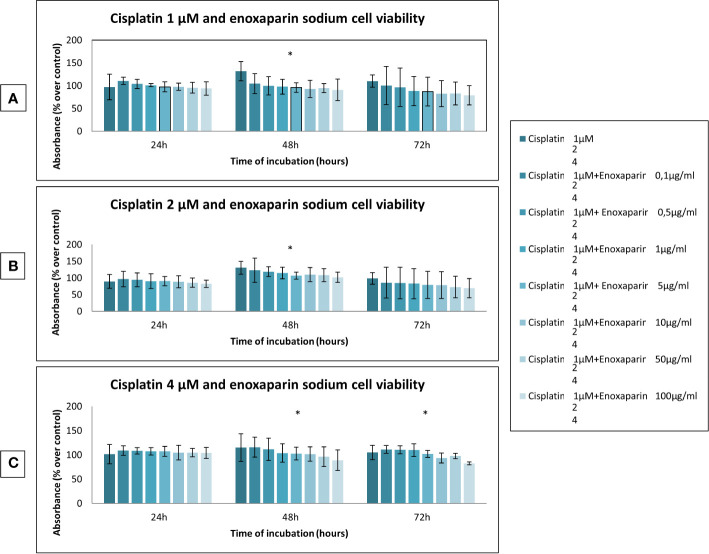
Effects of cisplatin (1, 2, and 4 µM) and enoxaparin sodium (0.1, 0.5, 1, 5, 10, 50, and 100 µg/ml) on H357 cell viability. **(A)** 24 h, *p* = 0.228; 48 h, *p* < 0.001; 72 h, *p* = 0.077. **(B)** 24 h, *p* = 0.729; 48 h, *p* = 0.006; 72 h, *p* = 0.502. **(C)** 24 h, *p* = 0.774; 48 h, *p* = 0.030; 72 h, *p* < 0.001. * means that there is significative differences at such picture.

**Figure 3:**
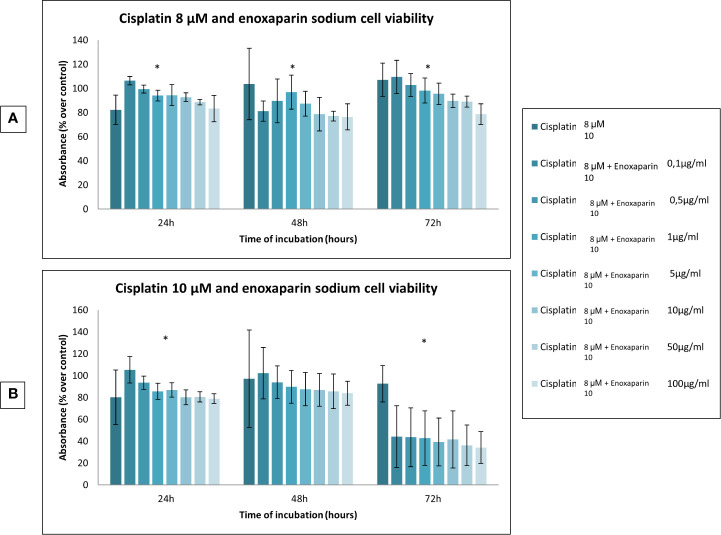
Effects of cisplatin (8 and 10 µM) and enoxaparin sodium (0.1, 0.5, 1, 5, 10, 50, and 100 µg/ml) on H357 cell viability. **(A)** 24 h, *p* < 0.001; 48 h, *p* < 0.001; 72 h, *p* < 0.001. **(B)** 24 h, *p* < 0.001; 48 h, *p* = 0.616; 72 h, *p* < 0.001. * means that there is significative differences at such picture.

When the different doses of cisplatin assayed (1, 2, 4, 8 and 10 µM) were combined with different concentrations of enoxaparin sodium (0.1, 0.5, 1, 5, 10, 50, and 100 µg/ml) it was found that combining any concentration of cisplatin with 100 µg/ml enoxaparin sodium produced the greatest synergic effect OSCC cell viability reduction, with statistically significant differences for combinations of 8 and 10 µM cisplatin at 24 h incubation (*p*<0.001 and *p<*0.001, respectively), and for 1, 2, 4 and 8 µM cisplatin at 48 h incubation (*p*<0.001, *p*=0.006, *p*=0.030, *p*<0.001, respectively) (**Figures 2** and **3**).

### Effects of Cisplatin and Enoxaparin Sodium on H357 Cell Apoptosis

Both the cell death test and the cell migration assay, investigated the two highest concentrations of cisplatin (8 and 10 µM), and enoxaparin sodium (100 µg/ml), as these doses led to the greatest reductions in cell viability.

**Figure 4:**
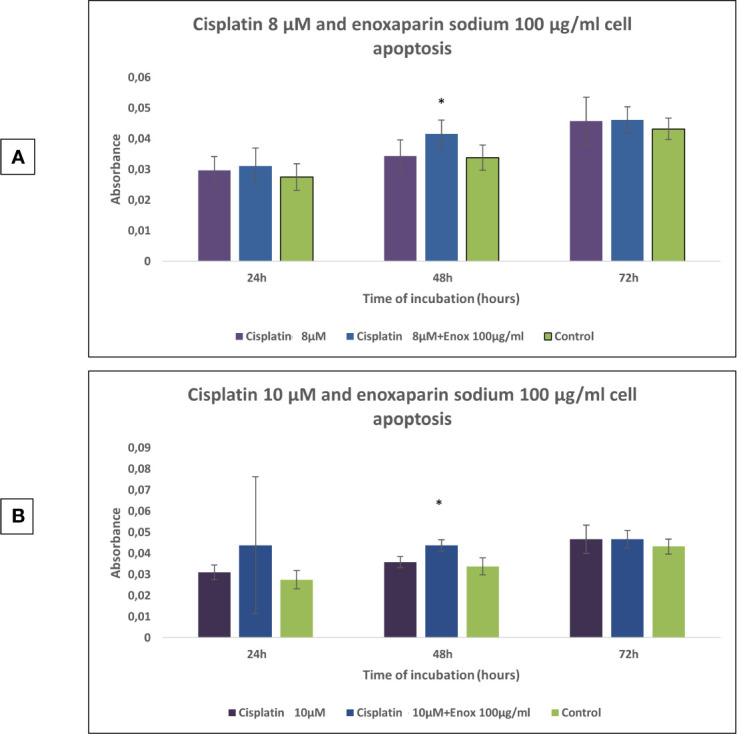
Effects of cisplatin (8 and 10 µM) and enoxaparin sodium 100 µg/ml on H357 cell apoptosis. **(A)** 24 h, *p* = 0.582; 48 h, *p* = 0.008; 72 h, *p* = 0.716. **(B)** 24 h, *p* = 0.413; 48 h, *p* = 0.009; 72 h, *p* = 0.592. * means that there is significative differences at such picture.

In the cell apoptosis test it was found that 24, 48, and 72 h incubation times all produced higher rates of apoptosis with the combination of 8 or 10 μM cisplatin and 100 μg/ml enoxaparin sodium, obtaining statistically significant differences at 48 h treatment (*p*=0.008 and *p*=0.009, respectively) (**Figure 4**).

### Effects of Cisplatin and Enoxaparin Sodium on H357 Cell Migration

**Figure 5:**
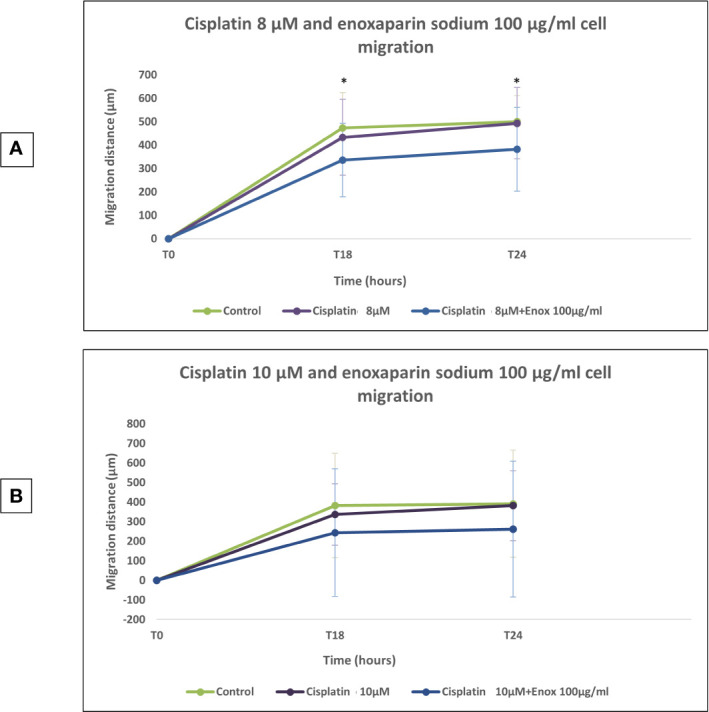
Effects of cisplatin (8 and 10 µM) and enoxaparin sodium 100 µg/ml on H357 cell migration. **(A)** 18 h, *p* = 0.003; 24 h, *p* = 0.004. **(B)** 18 h, *p* = 0.116; 24 h, *p* = 0.133. * means that there is significative differences at such picture.

**Figure 6:**
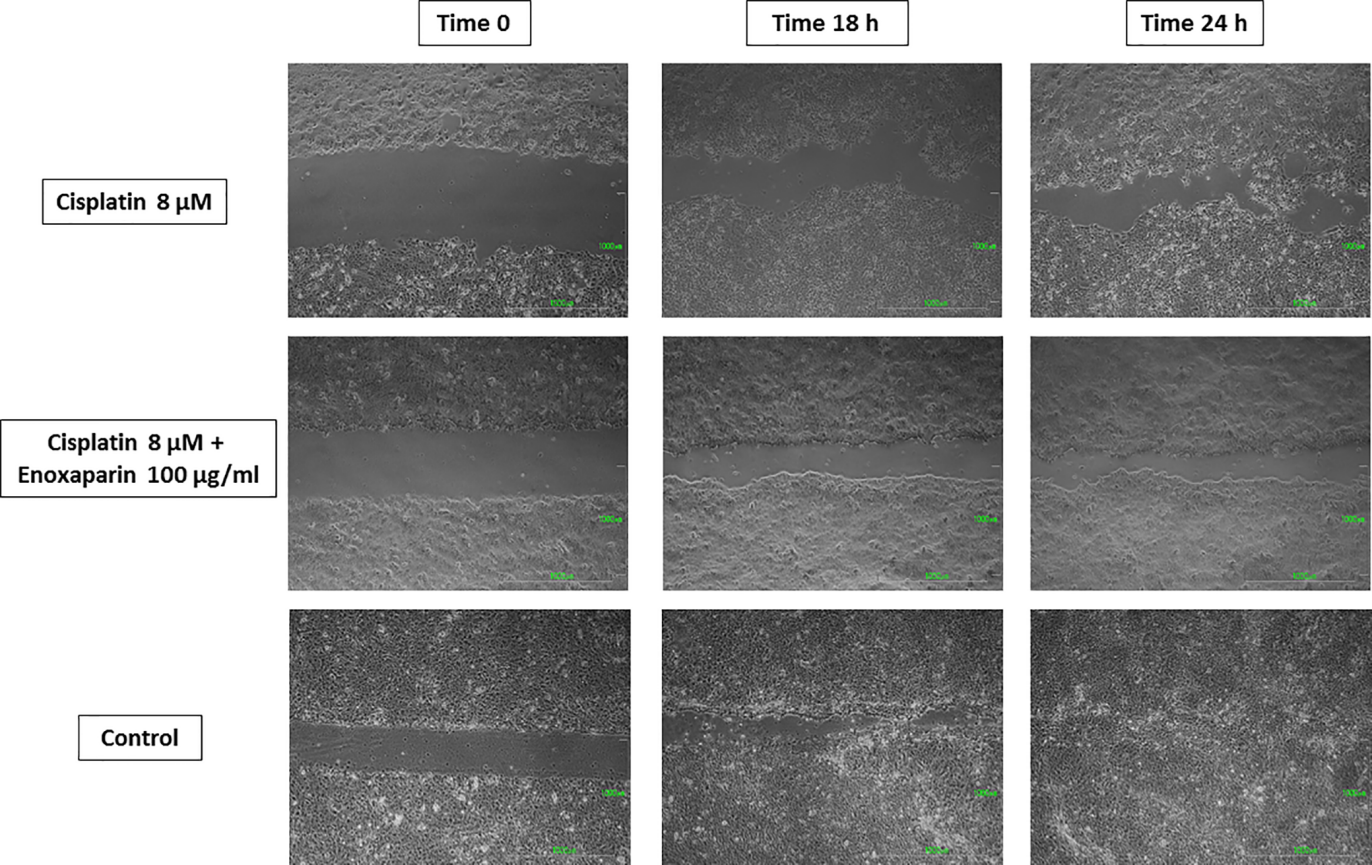
Cell migration into the wound space photographed at the time of initial wounding and at time intervals up to 18 and 24 h after wounding. Results of cisplatin 8 µM alone and combined with 100 µg/ml enoxaparin sodium.

**Figure 7:**
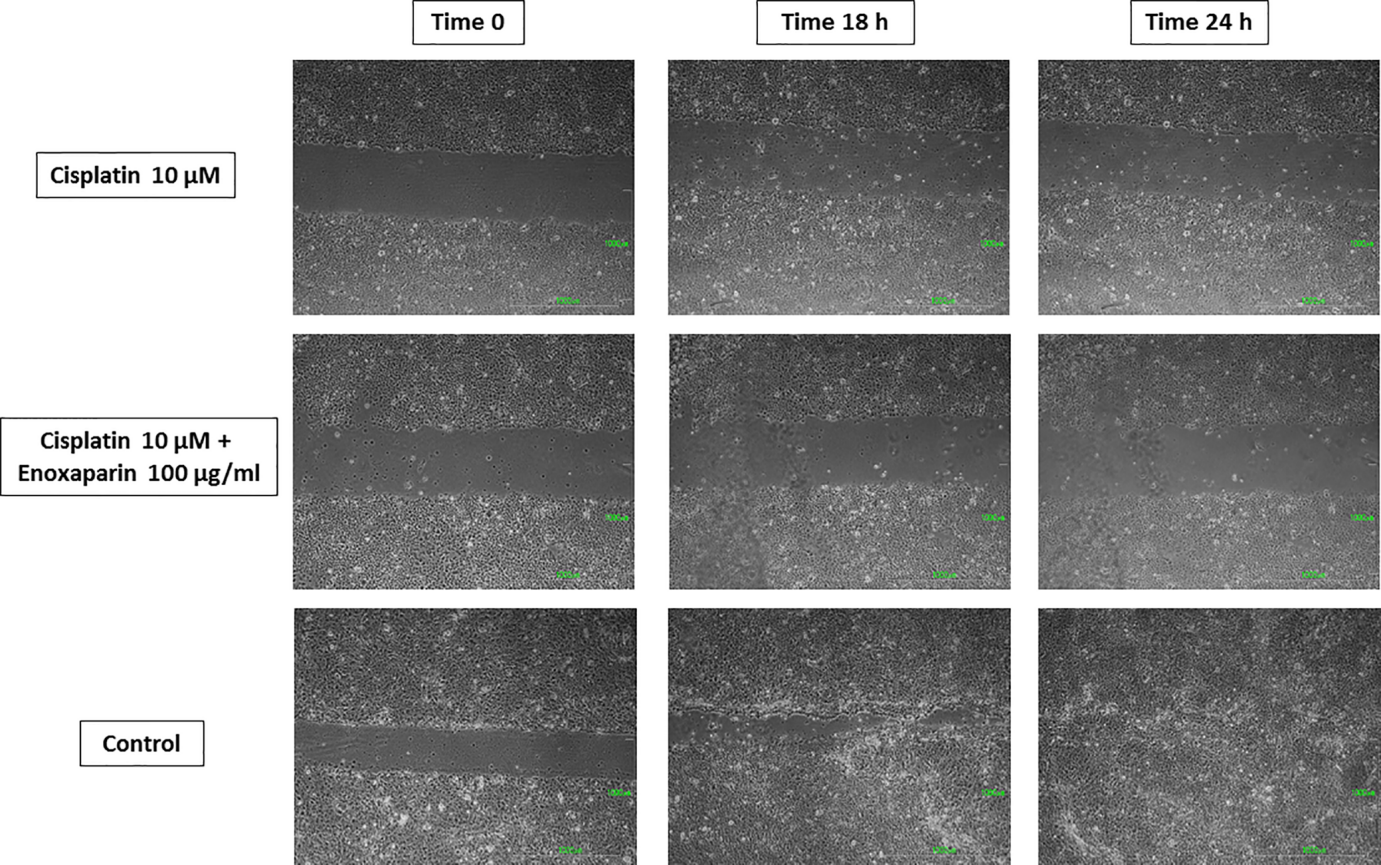
Cell migration into the wound space photographed at the time of initial wounding and at time intervals up to 18 and 24 h after wounding. Results of 10 µM cisplatin alone or combined with 100 µg/ml enoxaparin sodium.

When 8 or 10 μM cisplatin were combined with 100 μg/ml enoxaparin sodium, a greater reduction in cell migration capacity was observed, with statistically significant differences when 8 μM cisplatin were combined with 100 μg/ml enoxaparin sodium, both at 18 h (*p*=0.003) and 24 h (*p*=0.004) (**Figures 5**–**7**).

## Discussion

Most tumors in the oral cavity, pharynx and larynx (>90%) are squamous cell carcinomas. OSCC represents 6% of all malign neoplasias and constitutes the eighth most common cancer in terms of worldwide incidence (1). Mortality associated with OSCC remains high due to the fact that most cases are detected at an advanced stage, and also to treatment failure in the form of locoregional recurrence (15–50%) or distant metastasis (54, 55). The survival rate of patients with OSCC over 5 years is over 80% providing they receive treatment while the cancer is at an early stage. However, when the disease has spread to the cervical lymph nodes, this percentage decreases to 40%, and falls to only 20% when the case presents metastasis (56).

Cisplatin is the most often used chemotherapy in OSCC treatment, often administered in combination with taxanes and/or 5-fluoruracil (57). But in addition to the adverse effects of this drug (nausea, vomiting and toxic effects on different organs) (12), there are various routes by which the cancer can develop resistance to cisplatin’s anticarcinogenic action on OSCC: reduced formation of cisplatin-DNA adducts (which causes a reduction in cytotoxicity against carcinogenic cells) and generation of subpopulations of CSCs capable of ABC expression (drug transporter proteins) that may be responsible for OSCC resistance to cisplatin (23–25). In this context, development of an oral cancer-specific, anticancer drug is needed; new therapeutic strategies need to be identified and evaluated in preclinical models before entering clinical trials.

Heparin and LMWHs have shown substantial anticarcinogenic properties in addition to their traditional anticoagulant properties (34, 58, 59). It is possible that their anticarcinogenic action is due to: a) antiproliferative activity (due to their antiangiogenic activity) that impedes thrombin generation and so inhibits TF expression (41) and fibrin formation (42); and b) their antimetastatic activity deriving from their capacity for attachment to selectins, integrins, cytokines and enzymes such as heparanases (38–40). However, the action of LMWH, whether alone or in combination with cisplatin, on cell viability, apoptosis and cell migration capacity on human OSCC cells remains unknown.

The present study used enoxaparin sodium, which is an LMWH whose anticarcinogenic activity has been investigated inpancreatic adenocarcinoma cells, human breast carcinoma cells, human lung adenocarcinoma epithelial cells, glioma cells, melanoma cells (37, 43–47), and against metastasis of brain and colon cancer (48, 49) but never on human OSCC cells.

The present study assayed the effect of enoxaparin sodium on cell viability at 24, 48 and 72 h incubation, observing that, as the dose of LMWH increased, cell viability decreased, with the greatest reduction found with the 100 μg/ml dose, although no statistically significant differences were found at any of the incubation times assayed. Nevertheless, in 2011, Abu Arab et al. (45) observed an antiproliferative effect on human lung adenocarcinoma epithelial cell line A549 cultured with different concentrations of enoxaparin sodium (5, 10, 20, and 30 U/ml), obtaining statistically significant differences in comparison with a control group.

On the basis of the present results, it was found that cisplatin concentrations combined with 100 µg/ml enoxaparin sodium produced the greatest synergic effect on OSCC cell viability reduction, with statistically significant differences at concentrations of 8 and 10 µM cisplatin at 24 h incubation (*p*<0.001 and *p<*0.001, respectively), and for 1, 2, 4 and 8 µM cisplatin at 48 h incubation (*p*<0.001, *p*=0.006, *p*=0.030, and *p*<0.001, respectively). The present results for the action of cisplatin in combination with enoxaparin sodium up OSCC cells cannot be compared with any previous investigation of the possible synergic effects of these drugs for treating OSCC. Nevertheless, in 2016, Djaafar et al. (49) observed that enoxaparin sodium (200 μg/ml) reduced proto-oncogene regulator (cyclin D1) expression in mouse colon carcinoma cells MCA38. Cyclin D1 is related to the progression of G1 phase to S phase in the cell cycle. Its expression is generally increased in most tumors, but was seen to decrease through the action of enoxaparin sodium. Cell viability of colon cancer cells used in the study (MCA38) was seen to decrease after the reduction in cyclin D1 expression. This action of enoxaparin sodium combined with cisplatin’s action (whereby it induces apoptosis and arrest of the cell cycle resulting from its interaction with DNA, such as the formation of cisplatin-DNA adducts, which activate multiple signaling pathways) (8) could explain the synergic effect of the cisplatin/enoxaparin sodium combination on cell viability of the H357 human OSCC line.

When the action of cisplatin combined with enoxaparin sodium on cell apoptosis was assayed it was found that at 24-, 48-, and 72-h incubation times, higher rates of apoptosis were producedwhen treatment combined 8 or 10 μM cisplatin and 100 μg/ml enoxaparin sodium, obtaining statistically significant differences after 48 h treatment (*p*=0.008 and *p*=0.009, respectively). In 2006, Balzarotti et al. (47) obtained similar results, although these researchers investigated enoxaparin sodium alone, using primary cell cultures obtained from high-grade glioma; a statistically significant increase in cell apoptosis was produced with doses of 10 and 100 U/ml enoxaparin sodium in comparison with a control group. Recently, Niu et al. (29) have studied the possible synergic effect of another LMWH (Low-molecular weight heparin calcium) (Bopuquin, TianJing Chase Sun Pharmacological Co, Ltd, TianJing, China) on cell apoptosis in cisplatin-resistant and cisplatin-sensitive lung adenocarcinoma A459/DDP cells. The authors found statistically significant differences for both cell lines when cisplatin was applied combined with 5 IU/ml LMWH, compared with treatment by cisplatin alone and a control group.

Lastly, when 8 or 10 μM cisplatin were combined with 100 μg/ml enoxaparin sodium, this produced the greatest reduction in cell migration capacity, with statistically significant differences for 8 μM cisplatin with 100 μg/ml enoxaparin sodium, at both 18 h (*p*=0.003) and 24 h (*p*=0.004) incubation. The interaction of enoxaparin sodium with heparanase at the start of the tumor metastasis process would appear to be closely related to the phenomenon of reduction in cell migration. During this step in the process, carcinogenic cells degrade the extracellular matrix and the basal membrane (including its main components—heparan sulfate proteoglycans [PGHS]) through heparanase, subsequently releasing cytokines, chemokines, and angiogenic growth factors [VEGF, bFGF]), so favoring angiogenesis, tumoral growth and metastasis. However, the reduction in heparanase expression (overexpressed in most human tumors) by the action of enoxaparin sodium will reduce this cell migration mechanism. In a study by Djaafar et al. (49), treatment of mouse colon carcinoma cells MCA38 with 200 μg/ml enoxaparin sodium, significantly reduced heparanase expression after 24 h by up to 50% (both ARN and proteins). Enoxaparin sodium’s mode of action on the extracellular matrix will slow the cancer’s invasion process (related to the action of heparanase) and could explain the results obtained in the present study. Mousa et al. (37) using the B16 melanoma mouse model of metastasis, found that a pre-tumor cell injection of enoxaparin sodium followed by daily doses (for 14 days) reduced lung tumor formation by 70%, with significant differences in comparison with an animal control group. The best enoxaparin sodium results were published by Seeholzer et al. (46) who studied 25 patients with advanced breast cancer, pointing to good clinical outlook for the use of this LMWH for treating cancer.

In conclusion, the combination of cisplatin and enoxaparin sodium showed a synergic effect in reducing cell viability and migration capacity and increased the apoptosis of H357 human OSCC cells. The present results suggest enoxaparin sodium could be beneficial in chemotherapy for OSCC patients. Further laboratory and clinical assays should be conducted to confirm and develop the present findings (see **Tables 1** and **2**).

**Table 1:** Effects of cisplatin or enoxaparin sodium on H357 cell viability (ANOVA test).

Treatment/Time point	Absorbance (% over control)	*p-value*
	mean ± SD^*^	
**Cisplatin/24 h**		0.009
1 µM	97.21 ± 27.91	
2 µM	89.45 ± 20.53	
4 µM	101.51 ± 19.82	
8 µM	82.26 ± 12.23	
10 µM	80.33 ± 24.92	
**Cisplatin/48 h**		0.001
1 µM	131.92 ± 21.19	
2 µM	130.52 ± 19.24	
4 µM	115.09 ± 28.51	
8 µM	103.69 ± 29.54	
10 µM	97.12 ± 44.61	
**Cisplatin/72 h**		0.003
1 µM	110.11 ± 13.66	
2 µM	98.65 ± 17.26	
4 µM	105.01 ± 14.81	
8 µM	107.13 ± 13.78	
10 µM	92.61 ± 16.64	
**Enoxaparin sodium/24 h**		0.215
0.1 µg/ml	114.08 ± 15.01	
0.5 µg/ml	112.89 ± 20.24	
1 µg/ml	112.57 ± 11.37	
5 µg/ml	110.33 ± 19.54	
10 µg/ml	105.32 ± 20.21	
50 µg/ml	102.83 ± 12.78	
100 µg/ml	96.07 ± 29.31	
**Enoxaparin sodium/48 h**		0.558
0.1 µg/ml	107.21 ± 18.36	
0.5 µg/ml	111.94 ± 21-06	
1 µg/ml	114.04 ± 21.57	
5 µg/ml	106.41 ± 23.36	
10 µg/ml	105.30 ± 16.59	
50 µg/ml	108.82 ± 13.61	
100 µg/ml	97.76 ± 23.72	
**Enoxaparin sodium/72 h**		0.303
0.1 µg/ml	126.71 ± 33.14	
0.5 µg/ml	122.77 ± 25.51	
1 µg/ml	113.84 ± 23.14	
5 µg/ml	109.31 ± 23.28	
10 µg/ml	109.25 ± 17.37	
50 µg/ml	108.98 ± 29.21	
100 µg/ml	106.18 ± 15.51	

*SD, standard deviation.

**Table 2:** Effects of cisplatin (8 and 10 µM) and enoxaparin sodium (0.1, 0.5, 1, 5, 10, 50, and 100 µg/ml) on H357 cell viability (ANOVA test).

Treatment/Time point	Absorbance (% over control)	*p-value*
	mean ± SD^*^	
**Cisplatin 8 µM/24 h**		<0.001
Cisplatin 8 µM	82.26 ± 12.23	
Cisplatin 8 µM + Enoxaparin sodium 0.1 µg/ml	106.42 ± 3.59	
Cisplatin 8 µM + Enoxaparin sodium 0.5 µg/ml	99.48 ± 3.32	
Cisplatin 8 µM + Enoxaparin sodium 1 µg/ml	94.04 ± 4.54	
Cisplatin 8 µM + Enoxaparin sodium 5 µg/ml	94.37 ± 8.69	
Cisplatin 8 µM + Enoxaparin sodium 10 µg/ml	92.37 ± 3.57	
Cisplatin 8 µM + Enoxaparin sodium 50 µg/ml	88.61 ± 2.16	
Cisplatin 8 µM + Enoxaparin sodium 100 µg/ml	83.26 ± 10.92	<0.001
**Cisplatin 8 µM/48 h**		
Cisplatin 8 µM	103.69. ± 29.54	
Cisplatin 8 µM + Enoxaparin sodium 0.1 µg/ml	81.13 ± 8.39	
Cisplatin 8 µM + Enoxaparin sodium 0.5 µg/ml	89.60 ± 18.11	
Cisplatin 8 µM + Enoxaparin sodium 1 µg/ml	96.82 ± 14.07	
Cisplatin 8 µM + Enoxaparin sodium 5 µg/ml	87.29 ± 10.33	
Cisplatin 8 µM + Enoxaparin sodium 10 µg/ml	78.65 ± 13.95	
Cisplatin 8 µM + Enoxaparin sodium 50 µg/ml	77.07 ± 4.09	
Cisplatin 8 µM + Enoxaparin sodium 100 µg/ml	76.39 ± 10.78	
**Cisplatin 8 µM/72 h**		<0.001
Cisplatin 8 µM	107.13 ± 13.78	
Cisplatin 8 µM + Enoxaparin sodium 0.1 µg/ml	109.57 ± 13.82	
Cisplatin 8 µM + Enoxaparin sodium 0.5 µg/ml	102.78 ± 9.52	
Cisplatin 8 µM + Enoxaparin sodium 1 µg/ml	98.25 ± 10.45	
Cisplatin 8 µM + Enoxaparin sodium 5 µg/ml	95.54 ± 8.92	
Cisplatin 8 µM + Enoxaparin sodium 10 µg/ml	89.68 ± 5.71	
Cisplatin 8 µM + Enoxaparin sodium 50 µg/ml	89.08 ± 4.61	
Cisplatin 8 µM + Enoxaparin sodium 100 µg/ml	78.61 ± 8.62	
**Cisplatin 10 µM/24 h**		<0.001
Cisplatin 10 µM	80.33 ± 24.92	
Cisplatin 10 µM + Enoxaparin sodium 0.1 µg/ml	105.32 ± 11.96	
Cisplatin 10 µM + Enoxaparin sodium 0.5 µg/ml	93.59 ± 6.11	
Cisplatin 10 µM + Enoxaparin sodium 1 µg/ml	85.58 ± 7.36	
Cisplatin 10 µM + Enoxaparin sodium 5 µg/ml	86.88 ± 6.57	
Cisplatin 10 µM + Enoxaparin sodium 10 µg/ml	80.21 ± 6.81	
Cisplatin 10 µM + Enoxaparin sodium 50 µg/ml	80.58 ± 4.65	
Cisplatin 10 µM + Enoxaparin sodium 100 µg/ml	78.92 ± 4.41	
**Cisplatin 10 µM/48 h**		0.616
Cisplatin 10 µM	97.12 ± 44.61	
Cisplatin 10 µM + Enoxaparin sodium 0.1 µg/ml	102.21 ± 23.52	
Cisplatin 10 µM + Enoxaparin sodium 0.5 µg/ml	93.97 ± 14.99	
Cisplatin 10 µM + Enoxaparin sodium 1 µg/ml	89.82 ± 14.93	
Cisplatin 10 µM + Enoxaparin sodium 5 µg/ml	87.65 ± 15.31	
Cisplatin 10 µM + Enoxaparin sodium 10 µg/ml	86.92 ± 14.91	
Cisplatin 10 µM + Enoxaparin sodium 50 µg/ml	85.71 ± 15.85	
Cisplatin 8 µM + Enoxaparin sodium 100 µg/ml	83.91 ± 10.98	
**Cisplatin 10 µM/72 h**		<0.001
Cisplatin 10 µM	92.61 ± 16.64	
Cisplatin 10 µM + Enoxaparin sodium 0.1 µg/ml	44.22 ± 28.11	
Cisplatin 10 µM + Enoxaparin sodium 0.5 µg/ml	43.71 ± 26.87	
Cisplatin 10 µM + Enoxaparin sodium 1 µg/ml	42.83 ± 24.83	
Cisplatin 10 µM + Enoxaparin sodium 5 µg/ml	39.31 ± 21.81	
Cisplatin 10 µM + Enoxaparin sodium 10 µg/ml	41.64 ± 26.09	
Cisplatin 10 µM + Enoxaparin sodium 50 µg/ml	36.36 ± 18.53	
Cisplatin 10 µM + Enoxaparin sodium 100 µg/ml	34.23 ± 14.57	

*SD, standard deviation.

## Data Availability Statement

The original contributions presented in the study are included in the article/supplementary material. Further inquiries can be directed to the corresponding author.

## Author Contributions

All authors have worked in an equal way in research duties for developing this work. The authors declare that there is no any conflict interest regarding this submission. All authors contributed to the article and approved the submitted version.

## Funding

The fee will be paid with the grant of University of Murcia provided to Department of Oral Surgery. This paper is partially supported by Ministerio de Ciencia, Innovación y Universidades grant number PGC2018-097198-B-I00 and Fundación Séneca de la Región de Murcia grant number 20783/PI/18.

## Conflict of Interest

The authors declare that the research was conducted in the absence of any commercial or financial relationships that could be construed as a potential conflict of interest.
